# Arterial stiffness in type 2 diabetes: determinants and indication of a discriminative value

**DOI:** 10.6061/clinics/2021/e2172

**Published:** 2021-02-16

**Authors:** Clara Italiano Monteiro, Rodrigo Polaquini Simões, Cássia Luz Goulart, Claudio Donisete da Silva, Audrey Borghi-Silva, Renata Gonçalves Mendes

**Affiliations:** I Laboratorio de Fisioterapia Cardiopulmunar (LACAP), Departamento de Fisioterapia, Universidade Federal de Sao Carlos, Sao Carlos, SP, BR; II Programa de Pos-Graduacao em Ciencias da Reabilitacao, Universidade Federal de Alfenas, Alfenas, MG, BR

**Keywords:** Arterial Stiffness, Type 2 Diabetes Mellitus, Risk Factors, Carotid-Femoral Pulse Wave Velocity

## Abstract

**OBJECTIVES:**

To identify the clinical discriminative value and determinants of arterial stiffness in individuals with type 2 diabetes mellitus (T2DM).

**METHODS:**

This prospective cohort study included 51 individuals (53.57±9.35 years) diagnosed with T2DM (stage glucose≥126 mg/dL; diagnostic time: 87.4±69.8 months). All participants underwent an initial evaluation of personal habits, medications, and history; arterial stiffness assessment by carotid-femoral pulse wave velocity (cfPWV) using SphygmoCor; and blood laboratory analysis. A statistical analysis was performed using SPSS software, and values of *p*≤0.05 were considered significant.

**RESULTS:**

A cut-off cfPWV value of 7.9 m/s was identified for T2DM [Sensitivity (SE): 90% and Specificity (SP): 80%]. A subgroup analysis revealed higher glycated hemoglobin (Hb1Ac) (*p*=0.006), obesity (*p*=0.036), and dyslipidemia (*p*=0.013) than those with cfPWV ≥7.9 m/s. Multivariate analysis identified higher stage glucose (*p*=0.04), Hb1Ac (*p*=0.04), hypertension (*p*=0.001), and dyslipidemia (*p*=0.01) as determinant factors of cfPWV; positive and significant correlation between cfPWV and glucose (r=0.62; *p*=0.0003) and Hb1Ac (r=0.55; *p*=0.0031).

**CONCLUSIONS:**

In T2DM, an indicator of the discriminative value of arterial stiffness was cfPWV of 7.9 m/s. Clinical findings and comorbidities, such as hypertension, glucose, poor glycemic control, and dyslipidemia, were associated with and were determinants of arterial stiffness in T2DM. Reinforcement of monitoring risk factors, such as hypertension, dyslipidemia, and glycemic control, seems to be essential to the process of arterial stiffening. Confirmation of this discriminative value in larger populations is recommended.

## INTRODUCTION

Arterial stiffness (AS), as measured by the gold standard carotid-femoral pulse wave velocity (cfPWV) (1), is a key parameter of vascular changes resulting from a complex interaction between structural and functional factors of the elastic artery wall (2). It is characterized by increased pulse wave velocity along the arterial tree, with prognostic importance for increased systolic load and decreased myocardial perfusion pressure (3,4).

Type 2 diabetes mellitus (T2DM) has been implicated in accelerated AS and increased risk of cardiovascular (CV) disease (5). Previous studies have demonstrated that glycemic control and glycated hemoglobin (Hb1Ac) could directly influence the process of AS (6). Chen et al. (7) showed a positive association between T2DM and Hb1Ac, concluding that early glycemic control is fundamental in preventing the development of AS. Yamamoto et al. (8) also concluded that glycemic control, even in the short term, could improve AS.

The formation of glycation end products has been associated with harmful cross-linking of collagen molecules within the artery wall (9). This pathological condition can confer a decrease in the rate of degradation and high resistance to enzymatic proteolysis, contributing to increased collagen content accelerated in some conditions, such as T2DM. Increased sympathetic tone and oxidative stress and reduced nitric oxide bioavailability have also been shown to be associated with loss of elasticity and changes in the type or structure of elastin and/or collagen of the arterial wall (5,10). The presence of additional chronic conditions such as dyslipidemia, hypertension, and obesity could have a significant impact on cfPWV (11-13).

Although some studies have sought to establish reference values for cfPWV for diverse populations, such as European (3,14), Asian (15), and Argentinian (16), individuals diagnosed with diabetes were excluded from analyses. The identification of specific values in individuals with diabetes may be useful for higher accuracy in estimating CV risk, risk neglect, and subclinical CV disease. Additionally, understanding clinical factors and comorbidities as contributions of AS could add value in risk assessment and target strategies of treatments.

Considering the clinical importance and accelerated process of AS in T2DM, this study aimed to identify the clinical discriminative value and determinants of AS in individuals with T2DM. We hypothesized that there is an indication of the discriminative value of AS in T2DM, and clinical findings and comorbidities are associated with and are determinant factors for AS.

## MATERIALS AND METHODS

### Study Design and Population

A cross-sectional, observational, descriptive study was conducted in a sample of 51 individuals of both sexes diagnosed with T2DM, aged between 35 and 70 years.

Inclusion criteria: Individuals diagnosed of both sexes with T2DM (glucose≥126 mg/dL) (17), aged 35 to 70 years, who agreed to participate in the study and signed the informed consent form.

Non-Inclusion criteria: Individuals with a history of clinically proven cardiopathy and/or through examinations, vascular surgery of the carotid, femoral, or aortic arteries, uncontrolled hypertension, cognitive disorders that interfere with the understanding of the experimental procedure, pregnant women, and users of illicit drugs.

Exclusion criteria: Factors potentially detrimental to the quality of the measurements or making the wave recording unreliable.

All participants read and signed a consent form. This study was conducted following the principles of the Helsinki Declaration. The study was approved by the Human Research Ethics Committee of the University (CAAE 90754318.0.0000.5504).

### Study Protocol

The project was disclosed through social communication in news and radio, as well as pamphlet distribution and individual approaches directed by the researcher from November 2018 to March 2019. The participants were invited to the laboratory once to undertake the initial evaluation and AS assessment and were instructed to avoid alcoholic beverages and exercise within 12h before measurement; large meals, caffeine, and smoking within 3h before measurement; and wear light clothing such as t-shirts, shorts, or leggings.

Initial evaluation: The personal data of each volunteer, comprising full name, address, age, weight, and height, were collected. The volunteers were also questioned about medications in use, family, medical history, and physical activity level. The level of physical activity was considered according to the American College of Sports and Medicine (ACSM) (18): individuals who practiced less than 30 min of moderate activity 5 days a week or intense physical activity for 20 min on 3 days a week were considered sedentary. Dyslipidemia and hypertension were considered based on previous clinical diagnosis, according to the Brazilian guidelines for each disease (19,20). Body mass index (BMI) above 30 kg/m^2^ was considered obesity (21).

### AS Assessment

Pulse waves were obtained transcutaneously using the SphygmoCor device (AtCor Medical Pty Ltd, Australia) with transducers in the topography of the right carotid and right femoral arteries. Measurements were taken after a 10-min rest with the participant in the supine position. To determine the cfPWV, two pressure-sensitive transducers were placed on the skin of the most prominent parts of the right common and right femoral carotid arteries. The software uses the R wave of the electrocardiogram (ECG) and the base of the pulse wave to calculate the time and the speed in m/s that the wave takes to go through this stretch, which is the distance traveled by the wave between the right carotid and right femoral arteries, considering a speed limit of 10 m/s. Two measurements were performed with no difference higher than 5% between them, and the mean of the two measurements represented the cfPWV (22).

Blood sample analysis: Clinical laboratory tests were performed after 12h of fasting. Total cholesterol, high-density lipoprotein cholesterol (HDL-C), low-density lipoprotein cholesterol (LDL-c), triglycerides (TG), glucose (mg/dL), and HbA1c levels were determined using standard methods in the clinical laboratory.

### Statistical Analysis

The calculated minimum sample size was 46 participants, and it was determined *a priori* using GPower software 3.1.9 (Brunsbüttel, Germany), considering the mean outcome of the variable cfPWV (23), mean difference, and standard deviation with *α*=0.05, *β*=80%, *d*=0.85, and 1:1 allocation ratio in the two-tailed test. Descriptive data are presented as mean and standard deviation. The Kolmogorov-Smirnov test verified the data distribution. The receiver operating characteristic curve (ROC) analyzed the optimal limit values selected for each age considering cfPWV according to the study of reference values for AS (3). The confidence interval (95% CI) was used to determine the ability of the clinical variables, with the lower limit being higher than 0.50. Subsequently, the cut-off points of the variables that obtained significant areas under the ROC curve were identified, with the respective values of sensitivity and specificity. The Student’s t-test was used to compare the mean values, and the Fisher’s exact test was used to compare categorical variables between the subgroups with values equal to or above and below the cut-off value established for cfPWV. Pearson’s correlation was used to investigate the relationship between cfPWV and glucose, and cfPWV and Hb1Ac. Multivariate regression analysis was also performed to identify determinants of cfPWV in patients with T2DM. All tests were performed using the Statistical Package for the Social Sciences (SPSS), and the values were accepted as *p*≤0.05.

## RESULTS

Fifty-three participants were initially recruited, and two were excluded because they had a diagnosis of prediabetes; therefore, 51 participants were included in the final sample of this study. Table 1 shows sample characterization subdivided into general features, risk factors, medications, and blood analysis. More than half the population was obese, diagnosed with hypertension and sedentary, and had noncontrolled glycemia, as indicated by an analysis of Hb1Ac.

**Figure 1:**
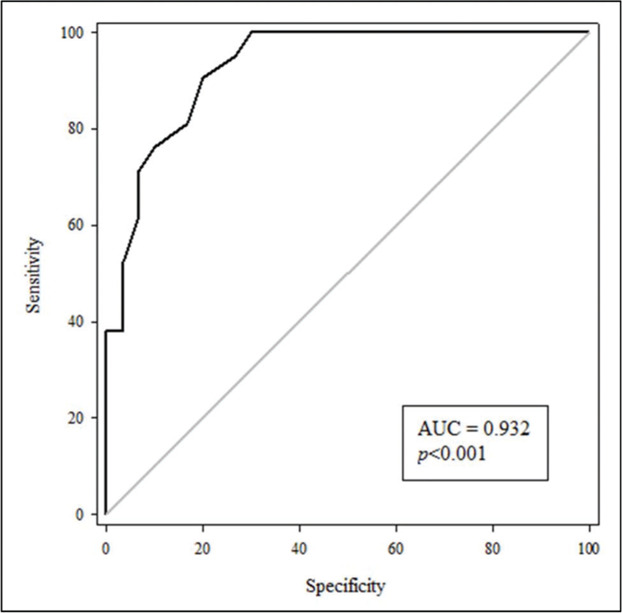
Receiver operating characteristic curve of cfPWV as an indicator of arterial stiffness for patients with type 2 diabetes mellitus. AUC: area under the curve; cfPWV: carotid-femoral pulse wave velocity.

To assess the usefulness of cfPWV as an indicator of AS for patients with T2DM, an ROC curve was constructed (Figure 1) with an area under the curve (AUC) value of 0.932 (*p*<0.001) with the program Sigma Plot 11.0. Moreover, a cfPWV of 7.9 m/s served as the cut-off point with a sensitivity of 90% and a specificity of 80%. The positive and negative values of the curve were 4.52 and 0.12, respectively.

Table 2 presents a comparison between a subgroup of patients with cfPWV<7.9 and cfPWV≥7.9 m/s, age, BMI, glucose, Hb1Ac, obesity, hypertension, sedentary status, and dyslipidemia. In the group with cfPWV values of ≥7.9 m/s, significantly higher values of Hb1Ac, obesity, and dyslipidemia were observed. Table 3 shows the results of the multivariate regression identifying the influence of clinical and comorbidities factors on cfPWV.

Pearson’s correlation demonstrated a positive and significant correlation between cfPWV and glucose (*r*=0.62, *p*=0.0003) and cfPWV and Hb1Ac (*r*=0.55, *p*=0.0031).

## DISCUSSION

The main findings of this study can be summarized as follows: I) A cfPWV cut-off of 7.9 m/s was established for individuals with T2DM as an indicator of AS in individuals with T2DM; (II) A subgroup with cfPWV ≥7.9 m/s presented higher values of Hb1Ac, obesity, and dyslipidemia than the group with cfPWV <7.9 m/s; III) positive and significant correlation between cfPWV and glucose and cfPWV and Hb1Ac; IV) Glucose, Hb1Ac, hypertension, and dyslipidemia were determinant factors of cfPWV in T2DM; and V) To the best of our knowledge, this study is the first to delineate an indication of the discriminative value of AS for individuals with T2DM.

It is well known that cfPWV is an important marker of AS and a predictor of cardiovascular risks (3,4). Although some studies have already reported cut-off values for general populations (11,13,16,24), they did not include individuals diagnosed with diabetes. These studies sought to establish a value for pulse wave velocity for their populations and evaluate the importance of assessing cfPWV in predicting cardiovascular events, thus contributing to a better understanding of AS.

Mendonça et al. (24) suggested cfPWV reference values for normotensive (9.11±0.16 m/s), controlled (9.12±0.18 m/s), and uncontrolled (9.42±2.2 m/s) hypertensive patients. In Argentina, Díaz et al. (16) reported a cfPWV value of 8.4 m/s for patients between 60 and 70 years old. In Uruguay, Farro et al. (25) reported a cfPWV value of 10.4 m/s for patients with hypertension under 60 years of age. In Brazil, for healthy patients aged 55 to 65 years, Baldo et al. (13) reported an average cfPWV of 9.48±1.39 m/s. Data from different European centers showed cfPWV values of 9.3 m/s and 11.1 m/s in elderly normotensive and hypertension patients, respectively (3).

According to Banegas and Townsend (26), the availability of cut-off or discriminative values is important for a better assessment of AS as a vascular biomarker, and its early detection is important in preventing the advancement of AS. In this way, these previous studies did not consider patients with T2DM, and although with a small sample, we were able to show a cfPWV cut-off value of 7.9 m/s for individuals with T2DM, which may be useful for higher accuracy in estimating CV risk.

As previously reported, individuals with diabetes present higher cfPWV values and higher risk for increased AS than those without diabetes, which is attributed to factors such as endothelial dysfunction, low-grade inflammation, and formation of advanced glycation end products in the vessel wall, resulting in loss of elasticity and intermolecular collagen cross-linking (27). These studies reinforce AS in the presence of diabetes; however, considering that arterial stiffness increased as defined in previous studies, for instance, cfPWV ≥12 m/s (28) could neglect actual potential risks and subsequent health care and complications.

Considering the presence of comorbidities in addition to T2DM, Lopes-Vicente et al. (27) explained that each risk factor, such as hypertension, hyperglycemia, and dyslipidemia, interferes independently through different mechanisms, causing negative changes in the function and structure of vessels. Moreover, our findings in multiple regression are in agreement with Gottsäter et al. (29) concerning the influence of hyperglycemia and dyslipidemia.

The negative impact of hypertension on AS is well established in the literature, and an interesting point to be highlighted is the favorable role of antihypertensive treatment for the reduction of AS, as demonstrated in the meta-analysis of Ye et al. (30). In our sample, 50% of the patients had a diagnosis of hypertension in the use of medication, and a comparative subanalysis between patients with and without hypertension revealed no statistically significant difference. Furthermore, although the number of patients with hypertension in the cfPWV ≥7.9 m/s group was not greater than that in the cfPWV <7.9 group (Table 2), hypertension was demonstrated to be a determining factor for AS. It is also noteworthy that hypertension and diabetes coexist in patients at a high prevalence (31), making the data closer to clinical reality.

Tolezani et al. (11) attested that the role of lipids in the properties of the great arteries has been widely discussed and remains controversial. The authors found that the lipid profile was a determinant of cfPWV and carotid distension. Some studies indicate that the relationship between cfPWV and lipid profile occurs through mechanisms such as the development of atherosclerotic plaques, oxidative stress, local and systemic inflammation, endothelial dysfunction, and low nitric oxide bioavailability (2,11). Thus, our findings regarding the influence of dyslipidemia on cfPWV are consistent; in addition, individuals with dyslipidemia had higher cfPWV values when subdivided according to a 7.9 m/s discriminative value, reinforcing the importance of this cut-off.

Regarding the correlation between cfPWV and glucose, cfPWV, and Hb1Ac, our findings are consistent with those of previous studies (6). Liang et al. (6) explained that high levels of circulating glucose in the long term leads to the formation of advanced glycation end products, resulting from the glycation of non-enzymatic proteins, creating irreversible cross-links in proteins of stable tissues. This may also explain the influence of glucose and Hb1Ac on AS, which we demonstrated in our multivariate regression. In addition, some studies indicate that the relationship between diabetes and elastic arteries arises early, even during a pre-diabetic state of insulin resistance (29).

Although some previous studies have demonstrated obesity as a potential determinant factor for AS (14), our study did not provide the same result. Our sample was composed of 50% of patients with obesity in class I (16), class II (8), and class III (2) by BMI (21). In a subgroup analysis, a greater number of patients with obesity (general) had cfPWV ≥7.9 m/s. In addition, it is important to assume that BMI measurement is an inaccurate measure of body fat content and, consequently, of healthy weight. Additional measures, such as waist circumference, have been recommended as more indicative of the risk of cardiovascular disease, which was not controlled in the present investigation.

Despite evidence of sex differences in AS, some results remain controversial, most likely due to the characteristics of the studied sample (32). One confounder factor could be the average age of females and males in these findings (33,34). Additionally, estrogen has been demonstrated to have a beneficial effect on the process of AS (35). The sex subgroup analysis according to the cfPWV cut-off demonstrated no difference, and 61% of females were in menopause and distributed similarly between groups.

There are some limitations to this study. First, the sample size was relatively small. Second, this cross-sectional study design did not provide information on the evolution of cfPWV over time, and it was not possible to insert evidence tracking of the phenomenon and coexistence of DM and comorbidities, although it was a more representative sample of a real population diagnosed with DM. Finally, we believe these results can significantly support professionals in clinical practice since AS has been widely recognized as an important reference for cardiovascular risk. We also reinforce the importance of the study, since it is the first to suggest an indication of discriminative value to AS for those diagnosed with diabetes and cfPWV≥7.9 m/s.

In conclusion, an indicative value of 7.9 m/s for cfPWV can be considered a reasonable cut-off for AS in patients diagnosed with T2DM. In our study, the identified value was also able to discriminate between clinical findings and comorbidities. Glucose, Hb1Ac, hypertension, and dyslipidemia were determinants of cfPWV in T2DM. Notably, a threshold is intended to simplify discrimination in clinical practice; however, the value is not the cut-off for binary identification of a patient's cardiovascular risk status due to its multifactorial nature. Therefore, there is reinforcement for preventive and continuous strategies for T2DM, focused on maintaining adequate glycemic levels as well as care for hypertension and dyslipidemia. Confirmation of this discriminative value in larger populations is recommended.

## AUTHOR CONTRIBUTIONS

All authors contributed to the study conception and design. Monteiro CI, Goulart CL and Silva CD were responsible for the material preparation, data collection, and analysis. The data analysis interpretation and the draft of the manuscript was performed by Monteiro CI with support and inputs from Simões RP, Goulart CL, Silva CD, Borghi-Silva A and Mendes RG. The supervision of the manuscript was performed by Mendes RG and Simões RP. All of the authors contributed to the final version of the manuscript.

## Figures and Tables

**Table 1:** Clinical, anthropometric, comorbidity characteristics, medications, and blood analysis in patients with T2DM.

General and clinical data	N=51
Age, years	53.57 (9.35)
Male, n (%)	28 (55)
Weight, kg	86.11 (16.91)
Height, m	1.69 (0.11)
Diagnostic time, months	87.4 (69.8)
BMI, kg/m^2^	30.46 (5.29)
BSP, mmHg	138.65 (19.43)
BDP, mmHg	82.49 (10.50)
PP, mmHg	41.69 (10.75)
MAP, mmHg	100.43 (13.29)
HR, bpm	70.43 (9.13)
cfPWV, m/s	9.17 (1.72)

Risk factors	
Obesity, n (%)	26 (50)
Obese class I, n (%)	16 (61)
Obese class II, n (%)	8 (31)
Obese class III, n (%)	2 (8)
Smoker, n (%)	5 (10)
Sedentary, n (%)	18 (35)
Hypertension, n (%)	26 (50)
Dyslipidemia, n (%)	47 (92)
Menopause, n (%)	14 (61)

Medications	
SGLT-2 inhibitors, n (%)	10 (20)
Sulfonylurea, n (%)	13 (25)
Biguanide, n (%)	15 (29)
Glyptin, n (%)	7 (14)
Insulin, n (%)	17 (33)
Diuretic, n (%)	8 (16)
Angiotensin-converting enzyme inhibitors, n (%)	2 (4)
Angiotensin II receptor blockers, n (%)	18 (35)
Beta-blockers, n (%)	7 (14)
Lipid reducer, n (%)	11 (20)

Blood analysis	
Insulin, mg/dL	14.18 (8.85)
Glucose, mg/dL	147.79 (51.60)
Hb1Ac, % [mmol/mol]	8.22 [66] (1.80)
HDL-c, mg/dL	45.92 (15.31)
LDL-c, mg/dL	103.09 (42.49)
Triglycerides, mg/dL	183.62 (128.20)
Total cholesterol, mg/dL	179.16 (45.99)

The data presented are described as mean (standard deviation) and in percentage of patients in the sample. BMI: body mass index; BSP: brachial systolic pressure; BDP: brachial diastolic pressure; PP: aortic pulse pressure; MAP: medium arterial pressure; HR: heart rate; cfPWV: carotid-femoral pulse wave velocity; SGLT-2: sodium/glucose cotransporter 2; Hb1Ac: glycated hemoglobin; HDL-c: high-density lipoprotein cholesterol; LDL-c: low-density lipoprotein cholesterol; T2DM: type 2 diabetes mellitus.

**Table 2:** Comparison of general data and risk factors between values equal to or above and below the cut-off value established for cfPWV.

	cfPWV <7.9 (n=23)	cfPWV ≥7.9 (n=28)	*p*
cfPWV	6.69 (0.71)	9.19 (1.77)	0.000
Age, years	53.78 (10.29)	53.39 (8.69)	0.886
BMI, kg/m^2^	29.04 (4.98)	31.67 (5.30)	0.076
Glucose, mg/dL	130.87 (29.10)	153.07 (35.23)	0.077
Hb1Ac, % [mmol/mol]	7.22 [55] (1.05)	8.97 [75] (1.85)	0.006
Obesity, n (%)	8 (35)	18 (64)	0.036
Hypertension, n (%)	14 (61)	14 (50)	0.438
Sedentary lifestyle, n (%)	7 (25)	12 (43)	0.361
Dyslipidemia, n (%)	6 (21)	17 (74)	0.013
Sex, female, n (%)	12 (52)	11 (39)	0.357

Data are presented as mean (standard deviation). cfPWV: carotid-to-femoral pulse wave velocity; BMI: body mass index; Hb1Ac: glycated hemoglobin.

**Table 3:** Multivariate regression analysis for the variables influencing the cfPWV.

Variables	Regression coefficient	95% CI	*p*
Age	0.029	-0.0256-0.0852	0.283
Glucose	0.001	4.0270-0.0004	0.046
Hb1Ac	-0.001	-0.0003-8.9682	**0.047**
BMI	0.132	-0.0329-0.2983	0.113
Obesity	0.154	-1.565-1.8133	0.852
Hypertension	1.736	0.6851-2.7877	**0.001**
Sedentary	-0.264	-1.2487-0.7192	0.596
Dyslipidemia	-1.181	-2.1317-0.2310	**0.016**

cfPWV: carotid-to-femoral pulse wave velocity; CI: confidence interval; Hb1Ac: glycated hemoglobin; BMI: body mass index.
